# Evaluation of the analytical performances of Cobas 6500 and Sysmex UN series automated urinalysis systems with manual microscopic particle counting

**DOI:** 10.11613/BM.2018.020712

**Published:** 2018-06-15

**Authors:** Ebubekir Bakan, Zafer Bayraktutan, Nurcan Kilic Baygutalp, Mehmet Ali Gul, Fatma Zuhal Umudum, Nuri Bakan

**Affiliations:** 1 Department of Medical Biochemistry, School of Medicine, Ataturk University, Erzurum, Turkey; 2 Department of Biochemistry, School of Pharmacy, Ataturk University, Erzurum, Turkey

**Keywords:** urinalysis, automated urinalysis, manual microscopy

## Abstract

**Introduction:**

Automated urinalysis systems are valuable tools in clinical laboratories, especially those with a high work load. The objective of this study was to compare the analytical performance of Sysmex UN series urine analyser, which may become a new one in our laboratory, with the Cobas 6500 automated urine analyser, which is used in our laboratory for a long time. For comparisons, manual microscopical examination was accepted as reference method.

**Materials and methods:**

A total of 470 urine samples were tested in the two automated urinalysis systems, and urine sediment testing with manual microscopy was applied to a 100 pathological samples of the total 470. The diagnostic performance of the two automated urine analysers was compared with each other and manual microscopy.

**Results:**

Differences were determined between automated and manual microscopy in some pathological samples. The resultant regression equations were as follows. Comparison of Cobas U701 with Sysmex UF-5000: y = - 0.57 (- 0.85 to - 0.29) + 0.95 (0.92 to 0.99) x for RBC, and y = - 0.11 (- 0.54 to 0.29) + 0.89 (0.84 to 0.98) x for WBC; comparison of Cobas U701 with manual microscopy: y = - 0.45 (- 0.85 to 0.21) + 1.00 (0.92 to 1.07) x for WBC; and comparison of Sysmex UF-5000 with manual microscopy: y = - 0.74 (- 1.09 to - 0.57) + 0.87 (0.85 to 0.91) x for WBC.

**Conclusions:**

We can conclude that the new Sysmex UN series urine analyser can be safely used in our laboratory. Although the results showed good to moderate concordance, the microscopy results of the automated platforms should be confirmed by manual microscopy, particularly in pathological samples.

## Introduction

Urinalysis constitutes a substantial part of routine laboratory analysis in clinical laboratories and plays an important role in hepatic, metabolic and haemolytic diseases, as well as the diagnosis and monitoring of kidney diseases and urinary tract infections (*1*, *2*).

Manual microscopic analysis of urine sediment is the ultimate analysis performed in clinical laboratories and is considered “the gold standard” for sediment analysis of urine. Unfortunately, manual microscopic urine analysis is not used effectively in clinical laboratories due to the lack of standardization of several steps of the analysis and this leads to inaccurate results (*3*). Since ensuring accuracy and repeatability for reliable urine analysis, particularly microscopic analysis, requires standardization, the National Committee for Clinical Laboratory Standards (NCCLS) and the European Urinalysis Guidelines recommend the use of automated urinalysis systems allowing quick and reliable results (*4*, *5*). Although some known disadvantages of automated urine microscopic analysers are still a point of debate, the advantages have led to widespread use in clinical laboratories as they provide improvements on the labour-intensive, time-consuming, wide-ranging and questionable manual microscopic analysis results (*6*).

Although the components of automated urinalysis platforms vary depending on the manufacturer, they are principally composed of two integrated main modules: the physical/chemical unit and the microscope unit (*7*, *8*). Many companies have developed new generation automated systems based on different technologies to automate urinalysis. Recently, Sysmex Corporation developed the Sysmex UN series, which is composed of three modules: UC-3500 (the chemical analysis unit), UF-5000 (the automated urine particle analyser part, which scans the formed element of urine with fluorescence flow cytometry), and UD-10 (digital particle screening device). The Cobas 6500 is another fully automated urine analyser, which has been developed by Roche Diagnostics and is currently being used in our laboratory.

The aim of this study was to evaluate the diagnostic performance of the new platform Sysmex UN series, which may become a new one in our laboratory, by comparing it with Cobas 6500 analyser, which is used in our laboratory for a long time, and manual microscopic examination. The manual microscopic examination was accepted as reference method for the evaluation of 100 pathological samples. Therefore, the results of the chemical and microscopic modules of the two analysers were compared, and the results of each microscopic module of the analysers were collated to those of manual microscopy in pathological samples.

## Materials and methods

### Materials

From the urine samples sent to our laboratory from out-patients and in-patients admitted to our hospital between April and May 2017, 470 samples were randomly selected and included in the study. Five to ten samples (approximately 8 samples) / day were randomly selected within the samples send to our laboratory. Pathological samples were not excluded from the study. We did not perform any inclusion or exclusion criteria on sampling in order to provide randomness. All procedures about collection, transport, preparation of specimens and urinalysis was performed according to European Urinalysis Guidelines (*5*). In brief, urine samples collected into a sterile primary container were immediately delivered to our laboratory and after transferring and aliquoting they were analysed within 1 hour, since the European Urinalysis Guidelines states that the time elapsing between voiding and examination of urine is a major obstacle to diagnostic accuracy in most laboratories. For urine sediment examination, samples were centrifuged in a refrigerated centrifuge for 5 minutes at 2480 x g.

There was no requirement for Ethics Committee approval or informed consent as the samples were sent to the laboratory for routine diagnostic purposes, and patient data privacy was ensured likewise the whole data routinely obtained from the laboratory automation system of our hospital.

All freshly collected urine samples taken from the patients were delivered to the laboratory in a sterile urine container of 120 mL volume (Becton Dickinson, New Jersey, USA).

Three parts of 8 mL samples each in BD tubes (Becton Dickinson, New Jersey, USA) of 11 mL volume were obtained from the original urine sample. No additives of preservatives were used in the portioning process as European Urinalysis Guidelines remarks that no preservatives are needed for most of the chemical constituents, which will be analysed, provided the analysis is performed within 24 hours and the tube has been refrigerated. Two portions were used for the automated urine analysis and one for manual microscopy. All evaluations of each sample were completed within a maximum of 1 hour. Before starting the analysis, to ensure internal quality control, between and within-run variations and carry-over measurements of workstations were evaluated with control materials. Liquicheck urinalysis control material level-2, which is commercially available (Biorad Laboratories, CA, USA), was used to provide analytical quality.

### Methods

#### Urinalysis systems

In this study, we aimed to evaluate the analytical performance of the new urine analyser included to our laboratory, Sysmex UN series, by comparing the results with the Cobas 6500 automated urine analyser, which is used in our laboratory for a long time. Both urinalysers report the microscopic results as cells/high power field (HPF) or low power field (LPF) in the same way as manual microscopy, making it easy to compare the devices and manual microscopy.

The Cobas 6500 platform is a combination of the Cobas u601 analyser (physical and chemical module) and the Cobas u701 analyser (microscopy module). The u601 analyser can evaluate the following parameters: pH, leukocytes, nitrite, protein, glucose, ketones, urobilinogen, bilirubin, erythrocytes, colour and clarity by reflectance photometry technology and specific gravity by refractometry. The Cobas u701 is a fully automated microscopy system for quantitative particle counting and semi-quantitative or qualitative classification of particles. Cobas u701 re-suspends the samples and pipettes them into disposable cuvettes, then centrifuges the cuvettes at 4409 x g for 10 seconds. A microscopic camera captures 15 real images of each centrifuged sample. Particle recognition software analyses the particles and the operator can see the sediment images on the screen. Cobas u701 can evaluate the following parameters: erythrocytes, leukocytes, hyaline cells, epithelial cells, bacteria, small round cells or no squamous epithelial cells, pathological casts, crystals, mucus, yeasts, and spermatozoa.

The Sysmex UN series is a combination of the UC-3500 analyser (physical and chemical module) and the UF-5000 analyser (microscopy module). Urine is analysed with reflectance photometry technology by dropping chemically absorbed pads (strips) and the following parameters are evaluated in the UC-3500 module: pH, leukocytes, nitrite, protein, glucose, ketones, urobilinogen, bilirubin, blood, erythrocytes, colour, clarity by reflectance photometry technology, and specific gravity by refractometry. UF-5000 is the fully automated microscopy module of the Sysmex UN series and counts cells with the fluorescence flow cytometric method. In this method, the nucleic acid components of the formed elements, which have a nucleus, are marked with polymethylene markers, and those without a nucleus are coloured through their lipid/proteins. Identification software determines and quantifies the cells and particles and, the results are displayed on the screen. An additional third module, UD-10, completes the Symex UN series, and this is a fully automated urine particle digital imaging device, which captures images of urine, classifies the particles into 8 classes on the basis of their size, and gives a detailed view of urine particles to the user. UD-10 confirms abnormal results after particle analysis.

In order to analyse the consistency of the results, the Sysmex UN series quality assesment processes were performed based on current guidelines (*5*). To determine between- and within-run precisions, control materials at different levels produced by the manufacturers were used according to current guidelines (*5*). Control material measurements with the Sysmex UN series were repeated 20 times for both day-to-day- and within-day basis. Coefficients of variations (CV%) were calculated using the manufacturer’s control materials (QC-UK0013LL and QC-UK0013HL for low and high WBC and RBC counts). A carry-over test was also executed for the Sysmex UN series by testing two different levels of control materials a total of three times: as the sequence of level-1 – level-2 – level-1.

#### Microscopy examination

From the total of 470 urine samples, 100, which were reported as pathological by one analyser or both, were subjected to manual microscopic examination. To minimize interobserver variability, all samples were examined by the same technician with the same microscope. For manual microscopic analysis, 8 mL of well mixed urine was centrifuged 5 minutes at 2480 x g (Allegra x-30R, Beckman Coulter, USA). After centrifugation, the sediment content was re-suspended and then slides were prepared and examined under a CX21FS1 upright microscope (Olympus, Tokyo, Japan) (*4*, *5*). Magnification x100 (LPF) was used for the crystals and x400 (HPF) for WBC and RBC. The diagnostic accuracies of sediment analysis compared to the manual microscopy for both Cobas 6500 and Sysmex UN series were evaluated using the classification in the different categories defined in Table 1. The particles were counted per field and a semi-quantitative classification system was performed by giving numerical values within some ranges (*e.g.* 0 - 10 or 0 - 5) or some results were named as negative or positive according to the previous reports similar to this study (*9*-*11*). Manual microscopy was accepted as the reference method in all reports.

**Table 1:** Urine sediment categories for semi-quantitative reference values

**Parameter**	**Range**				
**(-)**	**(+)**			
	**Few**	**Moderate**	**High**	**Many**
**RBC (cells/HPF)**	0 - 4	5 - 10	11 - 20	21 – 50	≥ 51
**WBC (cells/HPF)**	0 - 4	5 - 10	11 - 20	21 – 50	≥ 51
**Epithelial cell (cells/LPF)**	Negative	Few	Moderate	High	-
**Crystal (cells/LPF)**	Negative	Positive	-	-	-
RBC - red blood cells. WBC - white blood cells. HPF - high power field (x 400). LPF - low power field (x 100).					

#### Statistical analysis

PASW Statistics for Windows, version 20.0 (SPSS Inc., Chicago, USA) and MedCalc Statistical Software, version 12, (MedCalc Software, Mariakerke, Belgium) were used for statistical analysis. For continuous set variables, Kolmogorov-Smirnov test was used to evaluate the normality of data. For variables presented in ordinal scale (*i.e.* semi-quantitative categories), Kolmogorov-Smirnov test was not performed and non-parametric tests were used. A value of P < 0.05 was accepted as statistically significant.

Clinical decision was used as a determinant in order to classify the results obtained by different methods. For instance, WBC results were classified as (0) when the WBC count was normal, as (*1*) when rare, as (*2*) when low, as (*3*) when high, and as (*4*) when very high.

The comparison of data according to the clinical decision was performed using the McNemar test. Gamma and Spearman correlation analyses were performed for the comparison of parametric data (gamma correlation analysis was used when the linearity criteria of Passing-Bablok regression analysis were met and Spearman’s correlation analysis was used when it was not met). We accepted manual method as control and the analysed method (Sysmex UF-5000 or Cobas u701) as case when evaluating the concordance by gamma correlation analysis.

Concordance correlation analysis was used for the measurements of precision and accuracy. Passing-Bablok regression analysis was performed and Bland-Altman plots were used to demonstrate agreements and differences between the two automated analysers (*12*, *13*). When performing Bland-Altman graphs, we entered a value “4 cells” for the maximum allowed difference between methods selected the option “Draw lines for 95% CI of limits of agreement” as recommended by Stöckl *et al.* (*14*). Concordance correlation analysis was used for the measurements of precision and accuracy (*15*). Cohen’s κ concordance coefficient was used to estimate the agreement between the manual and the automated microscopic analysis results.

## Results

The diagnostic accuracies of sediment analysis compared to the manual microscopy are presented in Table 2. The coefficients of variations are shown in Table 3. Results showed that Sysmex UN series had satisfactory intra- and inter-assay precisions, and no carryover was detected by testing the control materials. The intra- and inter-assay precisions of Cobas 6500 were also reported as satisfactory in our previous report (*16*). The diagnostic accuracies of sediment analysis for both systems, compared to manual microscopy, were satisfactory. However, the Sysmex UN series was slightly more sensitive and specific than the Cobas 6500 for both leukocyte and erythrocyte counts.

**Table 2:** Diagnostic accuracy of automated urine analysers compared to manual microscopy

	**Sensitivity (%)**	**Specificity (%)**	**PPV (%)**	**NPV (%)**
**WBC - Cobas 6500**	86.1	91.8	86.8	91.5
**RBC - Cobas 6500**	45.3	95.2	81.0	79.5
**WBC - Sysmex UN-Series**	98.2	98.2	98.2	98.9
**RBC - Sysmex UN-Series**	99.2	94.9	89.7	99.6
WBC - white blood cells. RBC - red blood cells. NPV - negative predictive value. PPV - positive predictive value. Manual urine sediment analysis was used as reference.				

**Table 3:** Inter- and intra-assay coefficient of variations for WBC and RBC counts in low and high control materials in Sysmex UF-5000

**Control**	**Target value (x 10^6^/L)**	**LL-UL of target value (x 10^6^/L)**	**Intra-assay CV%**	**Inter-assay CV%**
**WBC low control**	38.5	(19.2 - 57.8)	9.2	3.2
**WBC high control**	779.4	(623.5 - 935.3)	3.2	3.2
**RBC low control**	40.5	(20.2 - 60.8)	3.2	4.1
**RBC high control**	200.2	(160.2 - 240.2)	6.2	3.2
WBC - white blood cells. RBC - red blood cells. CV - coefficient of variation. LL -lower limit. UL - upper limit.				

We reported correlation analysis results of RBC for the comparisons of manual microscopy *vs* Cobas u701 and manual microscopy *vs* Sysmex UF-5000 instead of Passing-Bablok regression analysis, since the CUSUM test for linearity resulted P < 0.05 and did not met the linearity criteria of Passing-Bablok regression analysis. Spearman’s correlation analysis results for RBC were as follows: comparison of manual microscopy *vs* Cobas u701: r = 0.76, P = 0.010 and manual microscopy *vs* Sysmex UF-5000: r = 0.98, P = 0.010. We reported Passing-Bablok regression analysis results for RBC comparisons of Cobas u701 *vs* Sysmex UF-5000, since the CUSUM test for linearity resulted P > 0.05 and met the linearity criteria of Passing-Bablok regression analysis.

**Figure 1:**
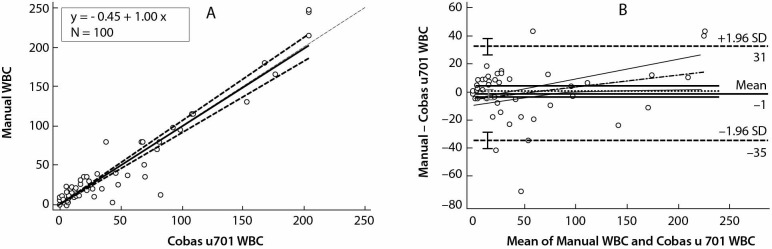
Comparison of manual analysis and Cobas u701 for white blood cells (WBC). a) Passing-Bablok regression analysis demonstrates similar performance of manual microscopy and Cobas u701 for WBC count. The regression line equation is shown in the box. Solid line - regression line. Dashed lines - 95% CI for the regression line. Dotted line - identity line (X = Y). b) Comparison of Cobas u701 with manual microscopy for WBC count using Bland-Altman analysis. Solid line (mean) – mean difference. Dashed lines (SD) – standard deviation. (B)

**Figure 2:**
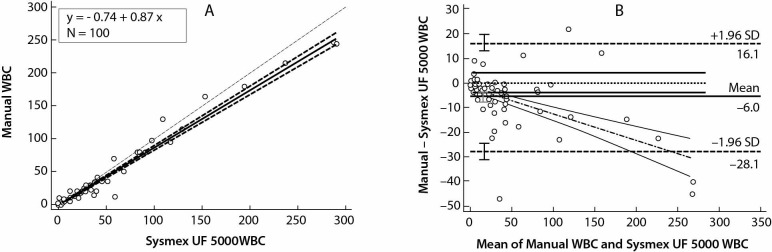
Comparison of manual analysis and Sysmex UF 5000 for white blood cells (WBC). a) Passing-Bablok regression analysis demonstrates similar performance of manual microscopy and Sysmex UF-5000 for WBC count. The regression line equation is shown in the box. Solid line - regression line. Dashed lines - 95% CI for the regression line. Dotted line - identity line (X = Y). b) Comparison of Sysmex UF-5000 with manual microscopy for WBC count using Bland-Altman analysis. Solid line (mean) – mean difference. Dashed lines (SD) – standard deviation. (B)

**Figure 3:**
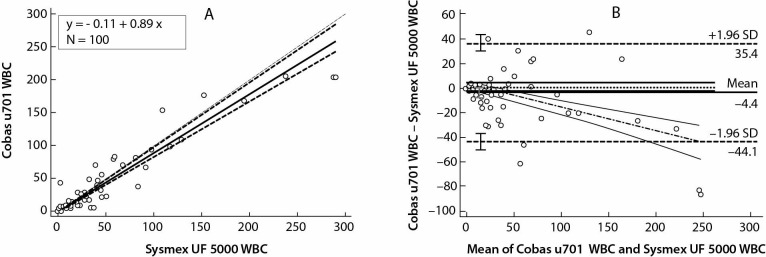
Comparison of Cobas u701 and Sysmex UF 5000 for white blood cells (WBC). a) Passing-Bablok regression analysis demonstrates similar performance of Cobas u701 and Sysmex UF-5000 for WBC count. The regression line equation is shown in the box. Solid line - regression line. Dashed lines - 95% CI for the regression line. Dotted line - identity line (X = Y). b) Comparison of Cobas u701 with Sysmex UF-5000 for WBC count using Bland-Altman analysis. Solid line (mean) – mean difference. Dashed lines (SD) – standard deviation. (B)

**Figure 4:**
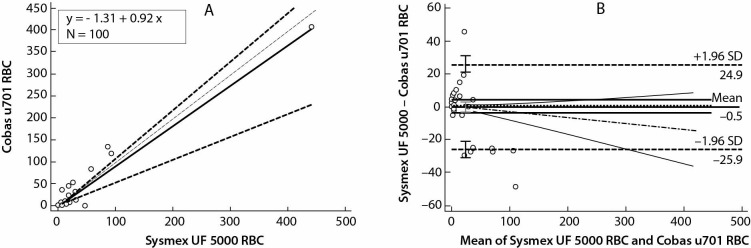
Comparison of Cobas u701 and Sysmex UF 5000 for red blood cells (RBC). a) Passing-Bablok regression analysis demonstrates similar performance of Cobas u701 and Sysmex UF-5000 for RBC count. The regression line equation is shown in the box. Solid line - regression line. Dashed lines - 95% CI for the regression line. Dotted line - identity line (X = Y). b) Comparison of Cobas u701 with Sysmex UF-5000 for RBC count using Bland-Altman analysis. Solid line (mean) – mean difference. Dashed lines (SD) – standard deviation. (B)

The results of the Passing-Bablok regression analysis for WBC and RBC counts are shown in Table 4, and Passing-Bablok regression graphs and Bland-Altman plots in Figures 1-4, respectively. The scatter diagrams with regression line, the first result of Passing-Bablok regression, enabled the visual inspection of measured data and obvious agreement of fitted regression line and identity line.

**Table 4:** The results of the Passing-Bablok regression analysis and Bland Altman plots for WBC and RBC counts

**Analytes**	**Comp**	**Passing-Bablok regression analysis equation,** **y = A x (95% CI) + B (95% CI)**	**Concordance correlation analysis**				**Bland-Altman graphs**			
**Cusum (P)**	**CCC** **(95% CI)**	**r**	**Cb**	**P**	**Bias** **(%)**	**LoA LL** **(95% CI)**	**LoA UL** **(95% CI)**
**WBC**	1 *vs* 3	1.00 (0.92 to 1.07) x + (- 0.45) (- 0.85 to 0.21)	0.530	0.95 (0.93 to 0.96)	0.95	1.00	0.340	- 1.6	- 35.09 (- 40.90 to - 29.28)	31.82 (26.01 to 37.63)
	2 *vs* 3	0.87 (0.85 to 0.91) x + (- 0.74) (- 1.09 to - 0.57)	0.380	0.98 (0.97 to 0.98)	0.99	0.99	< 0.001	- 6.0	- 28.09 (- 31.92 to - 24.25)	16.12 (12.28 to 19.95)
	1 *vs* 2	0.89 (0.84 to 0.98) x + (- 0.11) (- 0.54 to 0.29)	0.700	0.95 (0.93 to 0.96)	0.95	1.00	0.034	- 4.4	- 44.15 (- 51.06 to - 37.24)	35.45 (28.54 to 42.35)
**RBC**	1 *vs* 2	0.95 (0.92 to 0.99) x + (-0.57) (- 0.85 to - 0.29)	0.190	0.96 (0.94 to 0.97)	0.96	1.00	0.725	- 0.5	- 25.85 (- 30.26 to - 21.44)	24.94 (20.53 to 29.35)
Comp - comparisons: 1 - Cobas u701, 2 - Sysmex UF-5000, 3 - Manual microscopy. 95% CI – 95% confidence intervals. A – regression slope. B – regression intercept. Cusum - Cusum test for linearity. CCC – concordance correlation analysis. r - Pearson correlation coefficient. Cb - cias correction factor Cb (accuracy) (> 0.99 excellent agreement; 0.99 - 0.95 substantial agreement; 0.90 - 0.94 moderate agreement; < 0.9 poor agreement). LoA LL - lower limit of agreement. LoA UL: upper limit of agreement. WBC - white blood cells. RBC - red blood cells.										

Evaluation of Bland-Altman graphs showed that WBC and RBC counts showed acceptable comparability. According to the Bland-Altman plots, most paired data lay within 1.96 SD. No high mean biases for WBC were found in the current study.

For comparison between Cobas u701 *vs* manual, inspecting plots, intercept and slope values, we conclude that there is a constant difference between Cobas u701 *vs* manual (with a bias of - 1.6). Additionally, there are constant or proportional differences between Sysmex UF-5000 *vs* manual method for WBC and Sysmex UF-5000 *vs* Cobas u701 for RBC; there is a proportional difference between Sysmex UF-5000 *vs* Cobas u701 for WBC when we evaluated the 95% confidence intervals of intercept (A) and slope (B) for the aforementioned comparisons. We have to mention that the proportional CV of data might bias the analysis.

Passing-Bablok regression analysis showed very similar equivalent slopes. Most of the Passing-Bablok regression equations produced slopes of about 1.000 and intercepts of about 0.000 (Table 4). Only exceptions were the comparisons of WBC for Sysmex UF-5000 vs manual microscopy (slope did not contain one, intercept did not contain zero) and for Cobas u701 vs manual microscopy (slope did not contain one); and it was concluded that there were some significant differences between obtained intercept value and value zero, and there were no constant differences between two compared methods. Regression analysis demonstrated good concordance between manual microscopy which is used for the evaluation of 100 pathological samples, and Cobas u701 and Symex UF-5000. Similarly, the Bland-Altman difference plots demonstrated good compatibility between the three microscopic methods (Figures 1-3). Concordance correlation analysis showed substantial to excellent agreements.

WBC results were selected as an example to compare the microscopic results of the three methods (manual microscopy, Cobas u701, and Sysmex UF-5000) using gamma statistics and the McNemar test. The positivity and negativity rates of WBC counts of the three methods are given in Table 5. Gamma statistics were applied to evaluate the results in respect of clinical decision.

**Table 5:** Comparison of the numbers of WBC counted by the manual method and the urine analyzers

**Number of WBC (cells / HPF)**	**Number of WBC (cells / HPF)**						
**Cobas u701**						
**Manual Microscopy**		**0 - 4**	**5 - 10**	**11 - 20**	**21 - 50**	**≥ 51**	**Total**
	**0 - 4**	260	18	2	2	1	283
	**5 - 10**	21	21	9	1	0	52
	**11 - 20**	1	10	20	12	1	44
	**21 - 50**	0	2	5	18	7	32
	**≥ 51**	2	0	3	2	38	45
	**Total**	284	51	39	35	47	456
	**Sysmex UF-5000**						
**Manual Microscopy**		**0 - 4**	**5 - 10**	**11 - 20**	**21 - 50**	**≥ 51**	**Total**
	**0 - 4**	280	3	0	0	0	283
	**5 - 10**	3	43	5	1	0	52
	**11 - 20**	0	0	33	10	1	44
	**21 - 50**	0	0	0	30	2	32
	**≥ 51**	0	0	0	2	43	45
	**Total**	283	46	38	43	46	456
	**Cobas u701**						
**Sysmex** **UF-5000**		**0 - 4**	**5 - 10**	**11 - 20**	**21 - 50**	**≥ 51**	**Total**
	**0 - 4**	261	17	2	2	1	283
	**5 - 10**	19	19	8	0	0	46
	**11 - 20**	2	10	18	8	0	38
	**21 - 50**	1	5	9	22	6	43
	**≥ 51**	1	0	2	3	40	46
	**Total**	284	51	39	35	47	456
WBC - white blood cells. HPF - high power field (x 400).							

Comparison of Cobas u701 with manual microscopic particle counting method resulted a gamma statistics value of 0.958 (odds ratio 0.958, with a 95% confidence interval ranged from 0.517 to 1.773) and Mc-Nemar test presented significant difference between methods (P < 0.001) when cut-off value was accepted as < 5 WBC / HPF.

Gamma correlation showed 47 discordant pairs (case and control had different exposure to the risk factor). There were 24 (51.1%) pairs where the control was exposed to the risk factor but the case was not, and 23 (48.9%) pairs where the case was exposed to the risk factor but the control was not. The McNemar test demonstrated a significant difference in the comparison of Cobas u701 and the manual method at a ratio of 13.9%.

Comparison of Sysmex UF-5000 with manual microscopic particle counting method resulted a gamma statistics value of 0.99 and Mc-Nemar test presented significant difference between methods (P < 0.001). This comparison showed 6 discordant pairs (case and control had different exposure to the risk factor). There were 3 (50.0%) pairs where the control was exposed to the risk factor but the case was not, and 3 (50.0%) pairs where the case was exposed to the risk factor but the control was not. The two-tailed P value was determined as 0.6831, calculated with the McNemar test with continuity correction. The odds ratio was 1.000, with a 95% confidence interval ranging from 0.134 to 7.466 and non-concordance rate was 1.7%.

Comparison of Sysmex UF-5000 and Cobas u701 resulted a Gamma statistics value of 0.957 and Mc-Nemar test presented significant difference between the two automated methods (P < 0.001). This comparison showed 45 discordant pairs (case and control had different exposure to the risk factor). There were 23 (51.1%) pairs where the control was exposed to the risk factor but the case was not, and 22 (48.9%) pairs where the case was exposed to the risk factor but the control was not. The odds ratio was 0.957, with a 95% confidence interval ranging from 0.508 to 1.795 and non-concordance was 13.6%.

The κ analysis showed additional support for the comparisons of the couples: comparison of manual microscopic particle counting method and Cobas u701 resulted a κ value of 0.78 with P < 0.001, comparison of manual microscopy and Sysmex UF-5000 resulted a κ value of 0.97 with P < 0.001, and comparison of Sysmex UF-5000 and Cobas u701 resulted a κ value of 0.79 with P < 0.001 (κ = 0.79; P < 0.001).

## Discussion

Considering the Bland-Altman difference plots and the biases, we can conclude that the analytical performances of the two automated devices are acceptable. Gamma statistics indicated that the automated microscopic results of Cobas u701 and Sysmex UF-5000 and those of manual microscopy had very good to moderate compatibilities for WBC counts, which was confirmed by Passing-Bablok regression analysis and Bland-Altman plots. Gamma statistics for the WBC results showed a good to moderate correlation for the microscopic results of the three methods in respect of clinical decision. However, the McNemar test demonstrated a significant difference in the comparison of Cobas u701 and the manual method, showing that a non-concordancy at a rate of 13.9% may have affected all clinical diagnoses. This rate was very similar to that of the previous study by the current authors, which reported a non-concordance rate of 13.3% for the comparison of Cobas 6500 and Iris IQ200 fully-automatedurinalysers and comparison with manual microscopy (*17*). The McNemar test exhibited a significant difference in the comparison of Sysmex UF-5000 and the manual method (McNemar test P value < 0.001), and gamma correlation showed very low non-concordancy at a rate of 1.7%, which may have affected all clinical diagnoses only by this ratio. The Kappa analysis showed significant agreement when applied for the comparisons of manual and Cobas u701 (good agreement), manual and Sysmex UF-5000 (very good agreement), and Sysmex UF-5000 and Cobasu701 (good agreement).

Several microscopic comparison studies conducted to date have shown that automated analysers mostly have similar performances and that their results are compatible with manual microscopy. One of these studies, which was conducted by Akin *et al.* reported that both UriSed and IQ200 were highly reproducible and were able to analyse large numbers of urine samples quickly and simultaneously (*9*). Another study compared the diagnostic performance of three automated urinalysis systems (Iris IQ200, Sysmex UF-1000i and UriSed LabUMat) and demonstrated acceptable correlations in chemical data between instruments (*17*). Similar concordances have been reported in studies comparing LabUMat *vs* Urised and UX-2000 *vs* Cobas 6500 (*9*, *18*). A recent study conducted by Sánchez-Mora *et al.,* researchers aimed to compare UX2000 (Sysmex Corp, Japan) and SediMAX/AutionMax (Arkray Factory Inc., Japan), totally automatized analyzers, against Fuchs-Rosenthal counting chamber, the gold standard technique for sediment analysis. Reserachers reported that, UX-2000 has shown to have better concordance with the gold standard method compared to SediMAX/AutionMax. Researhcers suggested that Sysmex UX2000 needs some improvements such as an image module in order to decrease manual microscopy review for urine samples (*19*). Recently, we reported a comparison of Cobas 6500 and Iris IQ200 fully-automated urinalysers and a comparison of them with manual microscopy, which reported similar performances between the two autoanalyzers and good compatibility to manual microscopy (*16*). Several studies have reported that definition of WBC, RBC and epithelial cells are not always appropriate in automated urinalysers, especially in very pathological specimens (*9*, *10*, *20*).

Sysmex UN series automated urine analyser is very new on the market, and it is important to determine whether this platform is a candidate urinalyser in clinical laboratories. The current study is the first to compare the analytical performances of two automated urinalysers, the Cobas 6500 and Sysmex UN series.

It should be considered that although automated urinalysis systems have valuable advantages, they do not always report proper test results and controversial or pathological results should be carefully examined by the technician (*20*). In suspicious cases, the technician should carefully check the pre-analytical and analytical error sources. Analyzing urine sediment with manual microscopy in those situations is recommended (*9*, *10*, *16*). Moreover, the technician should examine the patient’s history and request that the test be repeated if necessary.

This study has several limitations. First, we selected urine samples randomly but only the samples over 8 mL were includedin the study and insufficient samples were excluded. Secondly, our results generally focus on the 100 pathologic samples of 470, and this number is relatively low for an appropriate comparison. Thirdly, although the negative and the positive predictive values ofthe two urinalysis system found in acceptable limits, these values could have been determinedmore sensitively if the KOVA cell chamber system had been used, since semi-quantitative results formanual microscopy were determined in this study. Moreover, chamber counting of uncentrifuged samples is the method of choice for instrument validation and, no sediment method can be considered as reference for quantitative urinary particle counting (*5*). Fourthly, we did not examine the specimen obtaining methods such as voiding, by catheterization, needle puncture, through a post-operative urostomy, urine collection bags or special receptables for bed-bound patients.

Despite these limitations, this study has the benefit that the measurements were obtained from the real patients and therefore the results can be applied directly to the clinical situation.

To the best of our knowledge this is the first study evaluating the analytical performance of Sysmex UN series by comparing that with Cobas 6500 and manual method. These two automated urine analysers showed good concordancies in most of the parameters analyzed. The comparison of the results with manual microscopy showed that the automated sediment analyzers have satisfactory analytical performances for formed elements. Therefore, we can conclude that the new Sysmex UN series urinalyser of our laboratory can be safely used in addition to Cobas 6500 automated urinalyser, which is being used in our laboratory for a long time.

Nevertheless, confirmation of pathological results with manual microscopy and consideration of the patients’ medical history is highly recommended.
